# Acute Treatment of Disabling and Nondisabling Minor Ischemic Stroke: Expert Guidance for Clinicians

**DOI:** 10.1161/STROKEAHA.125.053504

**Published:** 2025-12-11

**Authors:** Federico De Santis, Matteo Foschi, Lucio D’Anna, Shelagh B. Coutts, Urs Fischer, Pooja Khatri, Ahmed Nasreldein, Octávio Marques Pontes-Neto, Thanh N. Nguyen, Else Charlotte Sandset, Georgios Tsivgoulis, Guillaume Turc, Simona Sacco

**Affiliations:** Department of Biotechnological and Applied Clinical Sciences, University of L’Aquila, Italy (F.D.S., M.F., S.S.).; Department of Neuroscience, Neurology Unit–Stroke Unit, S. Maria delle Croci Hospital, Azienda Unità Sanitaria Locale (AUSL) Romagna, Ravenna, Italy (M.F.).; Department of Stroke and Neuroscience, Charing Cross Hospital, Imperial College London, National Health System (NHS) Healthcare Trust, United Kingdom (L.D.).; Department of Brain Sciences, Imperial College London, United Kingdom (L.D.).; Departments of Clinical Neurosciences, Radiology and Community Health Sciences, Hotchkiss Brain Institute, University of Calgary, Alberta, Canada (S.B.C.).; Department of Neurology, University Hospital Bern, University of Bern, Switzerland (U.F.).; Department of Neurology, Yale University, New Haven, CT (P.K.).; Department of Neurology, Assiut University Hospitals, Assiut University, Egypt (A.N.).; Neurology Division, Department of Neurosciences and Behavioral Sciences, Ribeirão Preto Medical School, University of São Paulo, Brazil (O.M.P.-N.).; Neurology Division, Radiology Division, Boston Medical Center, MA (T.N.N.).; Department of Neurology, Oslo University Hospital, Norway (E.C.S.).; Second Department of Neurology, National and Kapodistrian University of Athens, School of Medicine, “Attikon” University Hospital, Greece (G. Tsivgoulis).; Department of Neurology, Group Hospitalier Universitaire (GHU) Paris Psychiatrie et Neurosciences, Université Paris Cité, France (G. Turc).; Institute of Psychiatry and Neuroscience of Paris, Institut National de la Sanité et de la Recherche Médicale (INSERM) U1266, France (G. Turc).

**Keywords:** anticoagulants, ischemic attack, transient, ischemic stroke, thrombectomy, thrombolyic therapy

## Abstract

Minor ischemic strokes, usually defined as acute ischemic strokes with National Institutes of Health Stroke Scale score ≤5, account for over half of all cases and are often underestimated due to initially mild symptoms. Yet up to 30% of patients develop disability within 90 days, challenging the notion of a benign course. This guidance offers a pragmatic, scenario-based framework for acute minor ischemic stroke management, considering symptom severity (disabling versus nondisabling), eligibility for reperfusion, and presence of large vessel occlusion. Drawing from randomized trials, real-world evidence, and international guidelines, we examine therapeutic strategies, including dual antiplatelet therapy with aspirin plus a P2Y12 inhibitor, anticoagulation, intravenous thrombolysis, and endovascular treatment. Intravenous thrombolysis is preferred for disabling symptoms within 4.5 hours of symptom onset, whereas dual antiplatelet therapy remains standard for noncardioembolic, nondisabling events. For cardioembolic minor ischemic stroke ineligible for reperfusion, early anticoagulation within 48 hours appears safe and beneficial. Evidence for routine endovascular treatment in minor ischemic stroke with large vessel occlusion remains limited and controversial. We also address management of rapidly improving yet disabling symptoms and postreperfusion antithrombotic strategies, emphasizing individualized care and the need for further research.

Approximately 50% of patients with acute ischemic stroke present with minor neurological deficits.^1,2^ Despite the mild onset, up to 30% develop functional disability at 90 days,^3,4^ indicating that mild presentation does not guarantee benign outcomes. Acute minor ischemic stroke (MIS) poses specific therapeutic challenges. Clinical management must balance apparent benignity against risks of undertreatment or overtreatment, considering residual disability, early deterioration, recurrence, natural recovery, costs, and adverse events. This expert guidance offers a pragmatic, scenario-based approach to MIS management, aiming to support evidence-based decision-making and identify knowledge gaps for future research.

## Data Availability Statement

The authors declare that all supporting data are available within the article and its Supplemental Material.

## Ethics Statement

This expert guidance, based on published evidence and clinical practice, did not require ethical approval as no original patient data were used.

## Definition of MIS

**Figure 1.**
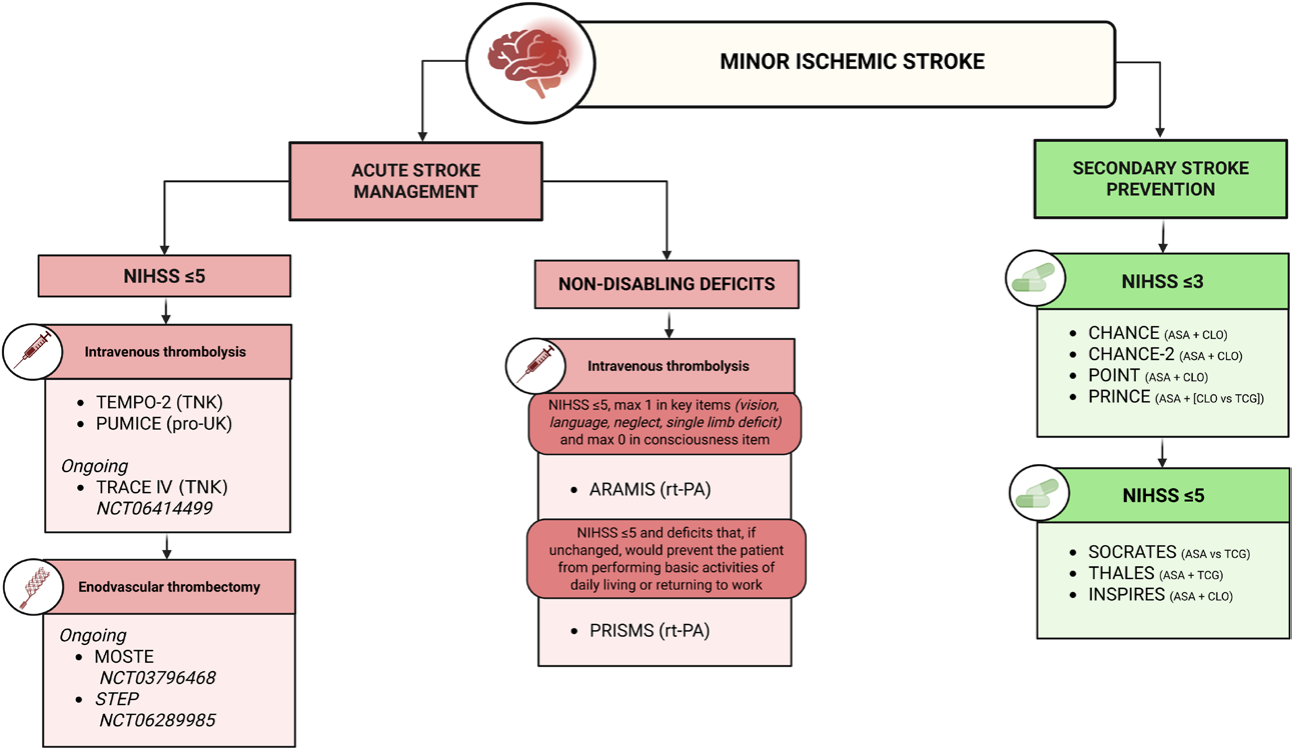
**Definitions of minor ischemic stroke across published and ongoing randomized controlled clinical trials.** ARAMIS indicates Antiplatelet Versus R-tPA for Acute Mild Ischemic Stroke; ASA, acetylsalicylic acid; CHANCE, Clopidogrel in High-Risk Patients With Acute Nondisabling Cerebrovascular Events; CHANCE-2, Ticagrelor or Clopidogrel With Aspirin in High-Risk Patients With Acute Nondisabling Cerebrovascular Events II; CLO, clopidogrel; INSPIRES, Intensive Statin and Antiplatelet Therapy for Acute High-Risk Intracranial or Extracranial Atherosclerosis; MOSTE, Minor Stroke Therapy Evaluation; NIHSS, National Institutes of Health Stroke Scale; POINT, Platelet-Oriented Inhibition in New TIA and Minor Ischemic Stroke; PRINCE, Platelet Reactivity in Acute Stroke or Transient Ischemic Attack; PRISMS, The Potential of rtPA for Ischemic Strokes With Mild Symptoms; pro-UK, prourokinase; PUMICE, Prourokinase Versus Standard Care for Patients With Mild Ischemic Stroke; rt-PA, recombinant tissue-type plasminogen activator; SOCRATES, Acute Stroke or Transient Ischemic Attack Treated With Aspirin or Ticagrelor and Patient Outcomes Trial; STEP, StrokeNet Thrombectomy Endovascular Platform; TCG, ticagrelor; TEMPO-2, Tenecteplase Versus Standard of Care for Minor Ischemic Stroke With Proven Occlusion; THALES, Acute Stroke or Transient Ischemic Attack Treated With Ticagrelor and Acetylsalicylic Acid for Prevention of Stroke and Death Trial; TNK, tenecteplase; and TRACE IV Tenecteplase Reperfusion Therapy in Acute Ischemic Cerebrovascular Events-IV.

MIS is defined by mild deficits, most commonly measured with the National Institutes of Health Stroke Scale (NIHSS; Figure 1). The most accepted cutoff is NIHSS score ≤5,^5–9^ although definitions vary widely.^10^ Some studies^11,12^ used stricter thresholds (≤3), whereas others^13–15^ included NIHSS score up to 10. Some define MIS by minimal involvement (0–1 point) in selected domains, excluding consciousness.^16^ For instance, ARAMIS (Antiplatelet Versus R-tPA for Acute Mild Ischemic Stroke)^17^ enrolled patients with NIHSS scores ≤5 and ≤1 point in vision, language, neglect, or limb weakness, and 0 for consciousness.

Guidelines^18,19^ apply flexible thresholds (NIHSS score ≤3 for clopidogrel+aspirin; ≤5 for ticagrelor+aspirin). Beyond numerical scoring, MIS is often classified as disabling or nondisabling.^17^ This distinction is context-dependent: aphasia, hemianopia, or mild limb weakness may be disabling despite a low NIHSS score, whereas isolated sensory loss or facial paresis are not. Disability perception varies by individual, and standardized criteria are lacking. Future trials should define disability using structured scales or prespecified syndromes and evaluate how such definitions affect outcomes.

## Practical Approaches to Acute Management of MIS

Treatment selection in MIS hinges on eligibility for reperfusion, degree of functional disability, and risk of deterioration if reperfusion is withheld. Functional impairment drives acute decisions: beyond obvious deficits such as aphasia or marked weakness, clinicians must detect subtler but meaningful problems—mild cognitive change, neglect, language disturbance, or gait ataxia—often underdetected by NIHSS yet impactful on autonomy and quality of life.

Although MIS commonly reflects small-artery occlusion, 10% to 20% show large vessel occlusion (LVO).^20,21^ Early neurological deterioration occurs in 8% to 30%,^7,22,23^ including ≈12% after intravenous thrombolysis (IVT),^24^ with a higher risk in carotid occlusion.^22^ Mechanisms include poor collaterals, thrombus propagation, evolving perfusion deficits,^25,26^ strategic lesion sites (brainstem, internal capsule),^27^ and comorbid cardiac, respiratory, or metabolic disorders. Early recurrence reaches up to 10% at 90 days^28–30^ and 1.9% to 5.2% at 7 days,^31–34^ especially with atherosclerosis or cardioembolism^35,36^ underscoring individualized secondary prevention. Goals are to restore function, prevent deterioration, and limit recurrence. Mortality is low (≈2% at 1 year^34–37^), and up to 30% of IVT-eligible patients recover spontaneously,^38,39^ so IVT benefits must be weighed against risks and costs, particularly in nondisabling MIS and low-resource settings. Current randomized controlled trials (RCTs)^17,40^ and meta-analyses^41^ show no clear IVT superiority over best medical therapy and suggest higher safety risks, with added complexity when LVO is present.

**Figure 2.**
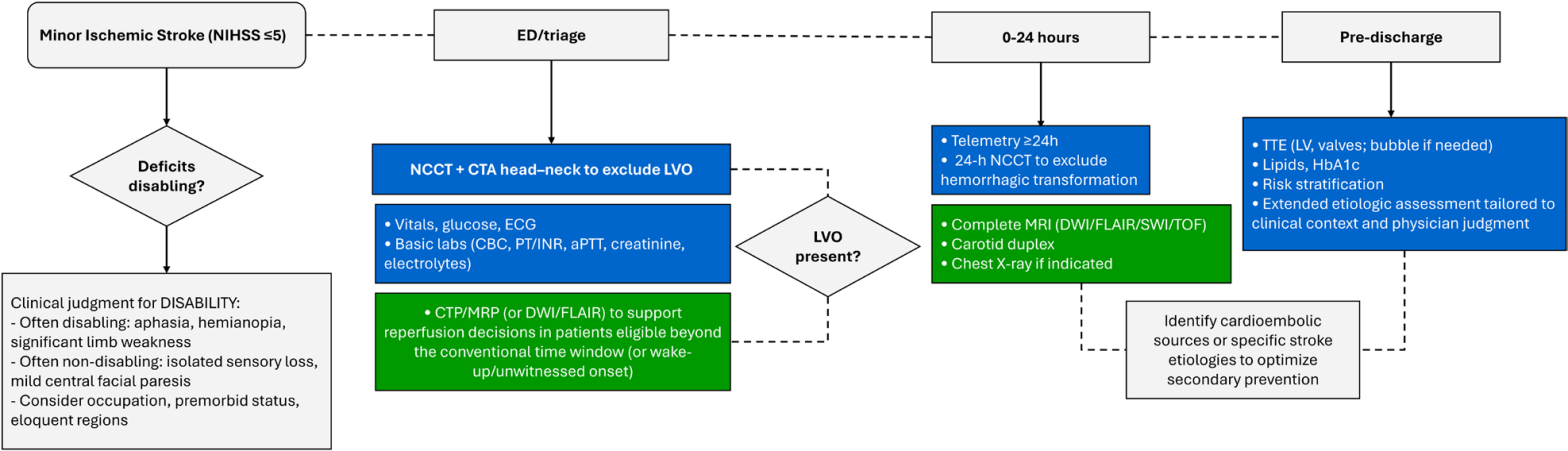
**Overview of the diagnostic work-up in the acute and subacute minor ischemic stroke setting, encompassing both mandatory and ancillary investigations.** Blue denotes mandatory tests; green, ancillary tests; diamonds, decision nodes; and rounded rectangle, entry point. aPTT indicates activated partial thromboplastin time; CBC, complete blood count; CTA, computed tomography angiography; CTP, computed tomography perfusion; DWI, diffusion-weighted imaging; ED, emergency department; FLAIR, fluid-attenuated inversion recovery; HbA1c, glycated hemoglobin; LV, left ventricular; LVO, large vessel occlusion; MRI, magnetic resonance imaging; MRP, magnetic resonance perfusion; NCCT, noncontrast computed tomography; NIHSS, National Institutes of Health Stroke Scale; PT/INR, prothrombin time/international normalized ratio; SWI, susceptibility-weighted imaging; TOF, time-of-flight; and TTE, transthoracic echocardiogram.

Optimal acute and subacute care requires comprehensive diagnostics (Figure 2). For this expert guidance, LVO is defined per RCTs criteria^42–44^ as intracranial internal carotid artery, M1 or proximal M2 middle cerebral artery, or basilar occlusion; anterior cerebral artery, posterior cerebral artery, and distal M2/M3 were inconsistently included and are not classified as LVO. Subsequent sections provide recommendations by disability, reperfusion eligibility, LVO status, symptom evolution, and prior therapy.

### Acute Minor Disabling Ischemic Stroke Without LVO, Eligible for IVT

Patients with acute MIS and disabling deficits without LVO form a clinically relevant subgroup. The main question is whether IVT should be offered despite mild presentation. An individual patient meta-analysis^45^ (6756 patients) showed consistent benefit of alteplase versus control across all severities, including NIHSS score 0 to 4 (n=666, odds ratio, 1.48 [95% CI, 1.07–2.06] for modified Rankin Scale [mRS] score 0–1 at 3–6 months). Accordingly, European Stroke Organisation (ESO)^46^ and American Heart Association/American Stroke Association^47^guidelines strongly recommend IVT (0.9 mg/kg alteplase) within 4.5 hours for disabling MIS.

Because IVT delays antiplatelet initiation, it may leave patients, especially those with symptomatic atherosclerosis, unprotected against early recurrence.^48–50^ Tenecteplase, a single-bolus alteplase analogue with greater fibrin specificity,^51^ proved noninferior in multiple RCTs^52–58^ and is endorsed by ESO^59^ as an equally effective, more practical alternative. A meta-analysis^60^ suggested slightly better 3-month outcomes without higher symptomatic intracranial hemorrhage (sICH^61^), though TEMPO-2 (Tenecteplase Versus Standard of Care for Minor Ischemic Stroke With Proven Occlusion),^7^ focused on MIS with occlusion ≤12 hours, found no benefit and increased bleeding and mortality, indicating possible harm in this subgroup. The strongest evidence supports IVT within 4.5 hours of symptom onset; data beyond this window are limited.^61^ EXTEND (Extending the Time for Thrombolysis in Emergency Neurological Deficits)^62^ (≤9 hours, perfusion-guided) and EXPECTS^63^ (Extending the Time Window for Thrombolysis in Posterior Circulation Stroke Without Early CT Signs; posterior strokes, ≤24 hours) showed improved independence, but their applicability to MIS remains uncertain.

**Figure 3.**
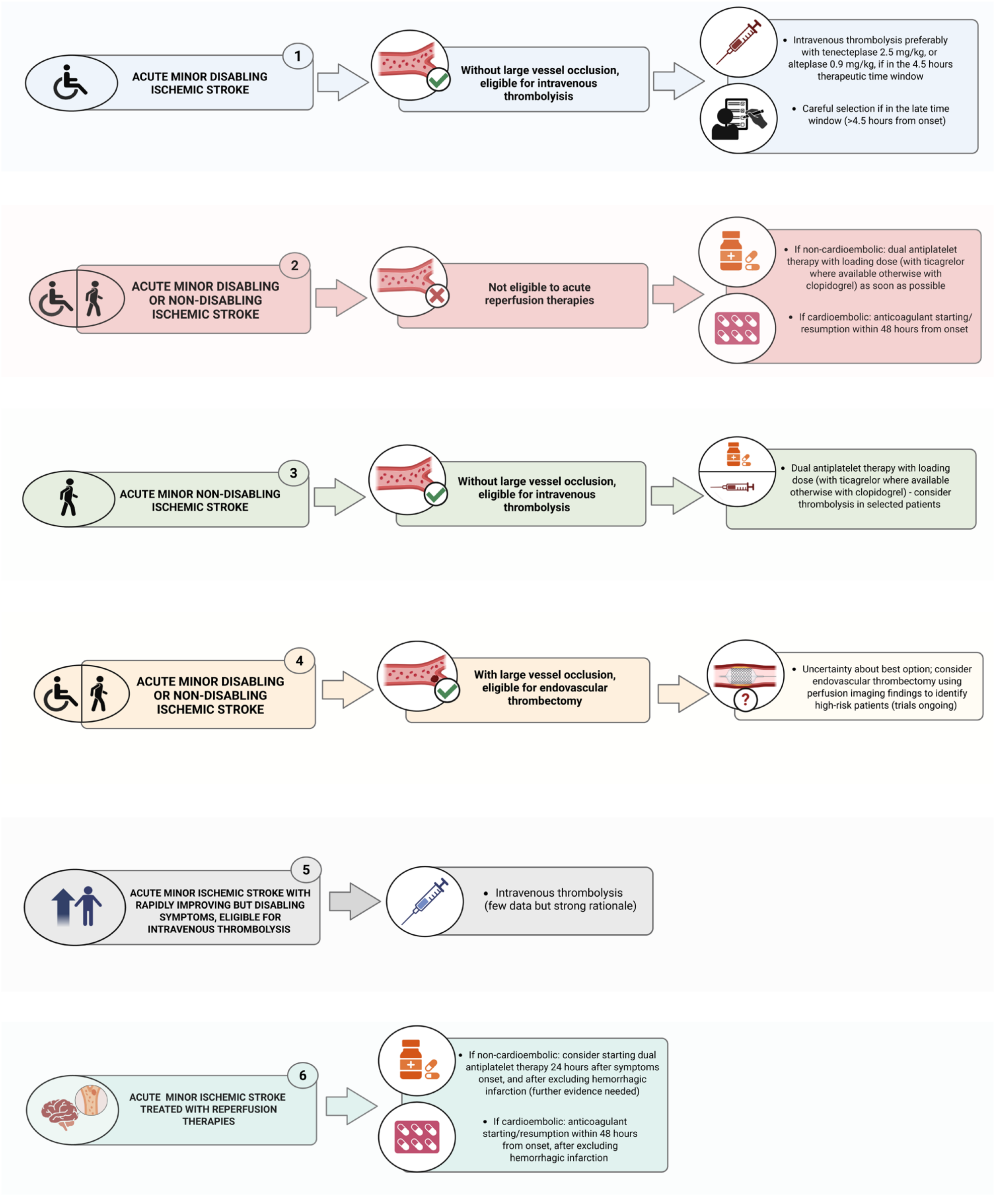
Management algorithm for patients with acute minor ischemic stroke across diverse clinical scenarios.

In summary (Figure 3), patients with disabling MIS should receive IVT within 4.5 hours (tenecteplase preferred, otherwise alteplase). Beyond this window or in a wake-up stroke, treatment should rely on perfusion or magnetic resonance imaging selection, though further evidence is needed. Dual antiplatelet therapy (DAPT; ie, aspirin combined with a P2Y12 inhibitor) remains the preferred option beyond the therapeutic window^6,11,12^ TRACE-IV (Tenecteplase Reperfusion Therapy in Acute Ischemic Cerebrovascular Events-IV; URL: https://www.clinicaltrials.gov; Unique identifier: NCT06414499) will compare tenecteplase versus DAPT in MIS (NIHSS score ≤5) and evaluate early post-IVT DAPT initiation to prevent recurrence. Table S1 summarizes supporting evidence for this scenario.

### Acute Minor Disabling or Nondisabling Ischemic Stroke, Not Eligible for Acute Reperfusion Therapies

Patients with acute minor disabling or nondisabling ischemic stroke who are not eligible for reperfusion (IVT, endovascular thrombectomy [EVT], or both) require careful antithrombotic selection. The key challenges are choosing between single antiplatelet and DAPT in noncardioembolic cases and defining optimal anticoagulation timing in cardioembolic strokes. The role of DAPT in acute noncardioembolic MIS has been evaluated in 4 pivotal RCTs^6,11,12,64^ (FASTER [Fast Assessment of Stroke and Transient Ischemic Attack to Prevent Early Recurrence], CHANCE [Clopidogrel in High-Risk Patients With Acute Nondisabling Cerebrovascular Events], POINT [Platelet-Oriented Inhibition in New TIA and Minor Ischemic Stroke], THALES [Acute Stroke or Transient Ischemic Attack Treated With Ticagrelor and Acetylsalicylic Acid for Prevention of Stroke and Death Trial]), which demonstrated that short-term DAPT with aspirin–clopidogrel or aspirin-ticagrelor effectively reduces early stroke recurrence, albeit with a modest increase in major bleeding.^65^ Based on this evidence, ESO^18^ and American Heart Association/American Stroke Association^19^ guidelines recommend 21 days of DAPT for MIS or high-risk transient ischemic attack within 24 hours of onset, followed by monotherapy. The INSPIRES trial (Intensive Statin and Antiplatelet Therapy for Acute High-Risk Intracranial or Extracranial Atherosclerosis)^66^ expanded this therapeutic window to 72 hours, confirming reduced recurrence but higher bleeding risk. Despite narrow inclusion criteria in RCTs, real-world data^67,68^ show that DAPT is widely used with similar effectiveness and acceptable safety profiles.

Clopidogrel efficacy depends on cytochrome P450 2C19 (CYP2C19) metabolism, and loss-of-function alleles—found in 25% of White, 30% of Black, and 60% of Asian patients^69,70^—reduce its antiplatelet effect. The CHANCE-2 trial (Ticagrelor or Clopidogrel With Aspirin in High-Risk Patients With Acute Nondisabling Cerebrovascular Events II)^71^ showed that ticagrelor–aspirin reduced 90-day stroke risk versus clopidogrel-aspirin (6.0% versus 7.6%, hazard ratio, 0.77, *P*=0.008) in carriers of these alleles, without more major bleeding. A Bayesian meta-analysis^72^ confirmed DAPT superiority over aspirin alone, ranking ticagrelor–aspirin highest for efficacy, although the difference disappeared when excluding CHANCE-2.

For cardioembolic MIS, the central issue is anticoagulation timing. Trials including TIMING (Timing of Oral Anticoagulant Therapy in Acute Ischemic Stroke With Atrial Fibrillation),^73^ ELAN (Early Versus Late Initiation of Direct Oral Anticoagulants in Post-Ischemic Stroke Patients With Atrial Fibrillation),^74^ OPTIMAS (Optimal Timing of Anticoagulation After Acute Ischemic Stroke With Atrial Fibrillation),^75^ and START (Optimal Delay Time to Initiate Anticoagulation After Ischemic Stroke in Atrial Fibrillation)^76^ —where MIS accounted for up to 58% of patients—found no interaction between baseline NIHSS score and timing of initiation. Given smaller infarct volumes and lower bleeding risk, patients with MIS are ideal candidates for early anticoagulation. The 1–3–6–12 days rule^77,78^ remains widely adopted. However, post hoc analyses from ELAN^79^ and the CATALYST (Collaboration on the Optimal Timing of Anticoagulation After Ischemic Stroke and Atrial Fibrillation: Prospective Individual Participant Data Meta-Analysis of Randomized Controlled Trials) meta-analysis^80^ showed that starting direct oral anticoagulants within 4 days reduced recurrent ischemic stroke without increasing the risk of sICH, including in patients with MIS (NIHSS score, 0–4).

In summary (Figure 3), patients with acute MIS not eligible for reperfusion should receive DAPT with loading doses for noncardioembolic stroke or early anticoagulation (within 48 hours) for cardioembolic origin. Clopidogrel-aspirin is the standard combination for DAPT, whereas ticagrelor–aspirin may be preferred for CYP2C19 loss-of-function carriers.^71^ THALES^6^ confirmed ticagrelor efficacy beyond Chinese cohorts,^11^ and its patent expiration may broaden access. For cardioembolic MIS, anticoagulation within 48 hours is recommended, given low bleeding risk; tirofiban evidence remains limited.^81–83^ Table S2 summarizes supporting data.

### Acute Minor Nondisabling Ischemic Stroke Without LVO Eligible for IVT

In patients with acute minor nondisabling ischemic stroke without LVO, management has long been debated, particularly regarding IVT versus antiplatelet therapy. Although reperfusion may appear unnecessary in patients with limited deficits, it was hypothesized to prevent early deterioration or subtle functional impairment.

Three major RCTs addressed this question. The PRISMS trial (The Potential of rtPA for Ischemic Strokes With Mild Symptoms)^40^ compared alteplase (0.9 mg/kg ≤3 hours) with aspirin (325 mg) in MIS (NIHSS score, 0–5) without disabling symptoms, showing no functional benefit (78.2% versus 81.5%) and higher sICH with alteplase (3.1% versus 0%), with <2% probability of meaningful benefit on Bayesian analysis.^40^ The ARAMIS trial^17^ compared DAPT (clopidogrel+aspirin for 12 days) with alteplase ≤4.5 hours, finding DAPT noninferior (93.8% versus 91.4% excellent outcomes) and safer (0.3% versus 0.9% sICH). Both trials lacked systematic angiography, leaving uncertainty about LVO prevalence. The PUMICE trial (Prourokinase Versus Standard Care for Patients With Mild Ischemic Stroke),^9^ testing prourokinase ≤4.5 hours versus antiplatelets, was stopped for futility (mRS score, 0–1: 73.5% versus 81.2%). Similarly, TEMPO-2^7^ found no benefit of tenecteplase ≤12 hours in MIS with intracranial occlusion or perfusion mismatch and observed higher mortality and sICH in the tenecteplase arm.

A Bayesian network meta-analysis^84^ identified DAPT as the most effective treatment for acute nondisabling stroke, with better outcomes than IVT, whereas another meta-analysis^85^ confirmed no functional advantage of IVT and higher sICH and mortality risk (Table S3). Based on these findings, ESO and American Heart Association/American Stroke Association guidelines^46,47^ recommend against IVT for acute nondisabling MIS within 4.5 hours, favoring early antiplatelet therapy. These recommendations are reinforced by PRISMS,^40^ ARAMIS,^17^ and TEMPO-2,^7^ along with meta-analyses^85^ and expert statements,^86^ establishing short-term DAPT as the standard of care. Nevertheless, some patients remain at risk of early neurological deterioration, particularly those with proximal arterial occlusions or long thrombi.^24^ Advanced neuroimaging can identify such high-risk features and guide individualized treatment.

In summary (Figure 3), early DAPT with clopidogrel-aspirin, or ticagrelor-aspirin when feasible, remains the standard for noncardioembolic, nondisabling MIS without LVO, providing a safer, equally effective alternative to IVT. IVT with tenecteplase, if available, or alteplase may be reserved for high-risk patients (eg, significant perfusion deficit or NIHSS score, 4–5^87^). Given population heterogeneity, larger RCTs are needed to guide management.^88^ Pending new evidence, DAPT—or anticoagulation when appropriate—should remain the reference approach in future studies.

### Acute Minor Disabling or Nondisabling Ischemic Stroke With LVO, Eligible for EVT

The role of EVT in patients with acute MIS and LVO remains debated. While EVT, alone or combined with IVT, is clearly superior to medical therapy in ischemic stroke with an NIHSS score ≥6, its benefit in MIS (NIHSS score, 0–5) is uncertain. Most MIS studies lacked systematic vascular imaging unless specifically designed for it (eg, TEMPO-2^7^). Population data^89^ suggest that ≈4% of patients presenting with NIHSS score <6 harbor LVO, and up to 20% may deteriorate without reperfusion.^90^ A meta-analysis^91^ of 11 observational studies (2019 EVT versus 3171 medical therapy) showed no functional advantage for EVT and higher 3-month sICH rates. These results were consistent across sensitivity analyses and indicated a possible benefit only in internal carotid artery or proximal M1 occlusions.^92^ However, all included studies were retrospective, limiting causal interpretation. Interestingly, IVT alone appeared to improve 3-month outcomes independently of EVT in patients with mild LVO.^93^

The influence of small vessel disease on EVT outcomes in LVO remains unclear. Some studies reported poorer outcomes and higher hemorrhagic risk,^94,95^ whereas others found no significant association.^96,97^ Accordingly, guidelines^98^ advise that small vessel disease should not affect acute management, even in MIS with or without LVO. Similarly, atrial fibrillation—found in only 21% to 24% of MIS cases^89–93^—may influence prognosis but not treatment selection.

American Heart Association/American Stroke Association,^47^ ESO,^99^ and Society of Vascular and Interventional Neurology (SVIN)^100^ guidelines recommend EVT for LVO with NIHSS score ≥6, whereas for MIS, they advise RCT enrollment comparing EVT plus medical therapy versus medical therapy alone. Because randomization is often unfeasible, ESO consensus^99^ considers EVT—with or without IVT—reasonable in patients with low NIHSS score with disabling deficits or post-IVT deterioration. The French MINOR-STROKE study^24^ identified proximal occlusion and long thrombus as predictors of early worsening, with a validated score aiding EVT selection. In the MINOR-STROKE–perfusion study,^87^ bridging therapy (IVT+EVT) led to worse 3-month outcomes and more hemorrhage in patients with small mismatch (≤40 mL), but similar outcomes when mismatch > 40 mL, suggesting perfusion-guided selection may optimize EVT use and should be confirmed in RCTs.

In summary (Figure 3), EVT cannot currently be recommended for unselected patients with MIS and LVO. It may be considered for those with low NIHSS scores but disabling symptoms (ie, isolated aphasia, homonymous hemianopia, or mild limb weakness that significantly impacts quality of life), large perfusion deficits, or clinical worsening despite IVT. Ongoing RCTs, including MOSTE (Minor Stroke Therapy Evaluation; URL: https://www.clinicaltrials.gov; Unique identifier: NCT03796468) and STEP (StrokeNet Thrombectomy Endovascular Platform; URL: https://www.clinicaltrials.gov; Unique identifier: NCT06289985, incorporating patients from ENDOLOW [Endovascular Therapy for Low NIHSS Ischemic Strokes] URL: https://www.clinicaltrials.gov; Unique identifier: NCT04167527), are expected to clarify the role of EVT in MIS with LVO.

### Acute MIS With Rapidly Improving but Disabling Symptoms, Eligible for IVT

Managing patients with acute MIS eligible for IVT but with rapidly improving symptoms and residual disability remains a clinical dilemma: whether to treat or adopt a conservative approach.

In the NINDS r-tPA trial (National Institute of Neurological Disorders and Stroke Recombinant Tissue Plasminogen Activator),^16,101^ exclusion for rapidly improving symptoms aimed to avoid treating transient ischemic attacks. However, patients with nonmild stroke who improve yet retain disabling deficits should not be excluded; IVT should proceed without delay. Both NINDS^101^ and ECASS III (European Cooperative Acute Stroke Study III)^102^ trials support IVT in improving but disabling cases, though neither focused on MIS.

A US registry^38^ of 29 200 patients with mild or improving symptoms not treated with IVT showed poor outcomes—28.3% not discharged home, 28.5% unable to walk independently—correlating with baseline NIHSS score. Similarly, Get With The Guidelines–Stroke data^103^ (42 394 cases) reported 27% not discharged home and 27.2% unable to ambulate independently, despite low mortality (0.8%).

A meta-analysis^104^ of 2905 MIS cases found early advantages in untreated patients but no 3-month differences (odds ratio, 0.99 [95% CI, 0.74–1.34]), likely due to indication bias, as IVT candidates typically had earlier arrival and better imaging. Among MIS with rapid improvement, IVT recipients had lower sICH (3.68% versus 5.77%) and mortality (odds ratio, 0.16 [95% CI, 0.09–0.31]) than patients with nonminor IVT. Conversely, untreated severe or LVO strokes had worse outcomes.^91^ Thus, bleeding risk must be weighed against residual disability or early deterioration if IVT is withheld. A post hoc ARAMIS analysis^105^ found DAPT (clopidogrel+aspirin) superior to IVT for nondisabling MIS without LVO but did not address residual disability.

In summary (Figure 3), although NINDS^101^ and ECASS III^102^ support IVT for improving yet disabling symptoms, no RCT has targeted MIS with rapid improvement and residual deficits. Management should be individualized based on residual disability, comorbidities, and cause.^106^ IVT may benefit patients at high risk of persistent disability—particularly nonlacunar strokes (large artery atherosclerosis, cardioembolism)—whereas lacunar MIS, often benign,^107^ may gain less. Large multicenter observational studies are warranted to clarify safety and guide future RCTs.

### Acute MIS Treated With Reperfusion Therapies

In patients with acute noncardioembolic MIS treated with IVT, EVT, or both, the optimal postacute antithrombotic regimen remains uncertain. Reperfusion aims to restore neurological function, whereas DAPT reduces early recurrence risk, but evidence on their combination is limited. Landmark DAPT trials^6,11,12^ excluded patients undergoing reperfusion, and safety concerns about increased intracranial hemorrhage or hemorrhagic transformation persist.

An observational study^108^ of 1373 patients with MIS compared IVT plus DAPT (aspirin combined with clopidogrel) with DAPT alone after propensity matching. Ninety-day favorable outcomes (mRS score, 0–2) were comparable, but ordinal mRS scores and early neurological improvement favored the combination, which also showed fewer recurrent vascular events (hazard ratio, 0.27 [95% CI, 0.08–0.90]) without excess bleeding. DAPT was usually started >24 hours after IVT and after excluding hemorrhage. A meta-analysis^109^ of 3 retrospective studies confirmed better 90-day outcomes without increased intracranial hemorrhage or mortality.

For cardioembolic MIS, optimal anticoagulation timing postreperfusion remains debated. Given the low hemorrhagic risk, delaying beyond 48 hours seems unnecessary. The ELAN trial^79^ (≈40% IVT, 20% EVT) showed early anticoagulation (<48 hours) reduced composite outcomes (2.9% versus 4.1%) versus delayed therapy, whereas CATALYST^80^ found direct oral anticoagulants within 4 days lowered recurrence without raising sICH risk, with the largest relative benefit in mild strokes.

In summary (Figure 3), for noncardioembolic MIS, early DAPT—initiated about 24 hours after onset once bleeding is excluded—can improve recovery and reduce recurrence without raising hemorrhagic risk. Single antiplatelet therapy suits low-risk or high-bleeding-risk patients, whereas short-term DAPT (≈21 days) benefits higher-risk ones. For cardioembolic MIS, early anticoagulation within 48 hours is safe and effective, as shown in ELAN^74,79^ and CATALYST.^80^ Further RCTs are warranted; the ongoing TAPIS trial (Ticagrelor With Aspirin Dual Antiplatelet Therapy Combined With Intravenous Thrombolysis for Ischemic Stroke; URL: https://www.clinicaltrials.gov; Unique identifier: NCT06316570) will assess early ticagrelor–aspirin DAPT after IVT.

## Treating MIS in Low-Resource Settings

Managing MIS in low-resource settings is challenging due to limited access to neuroimaging, thrombolytics, EVT, and stroke units.^110^ Resources often prioritize severe strokes or MIS with LVO eligible for reperfusion. Multimodal computed tomography (noncontrast computed tomography, computed tomography angiography, computed tomography perfusion) is available in only 27% of middle-income and even fewer low-income centers,^111^ making MIS with LVO difficult to diagnose. Clinical clues (fluctuating symptoms, cortical signs) and noncontrast computed tomography markers (insular ribbon loss, hyperdense middle cerebral artery) may raise suspicion, whereas ultrasound offers a low-cost alternative.

Given the high cost of thrombolytics and EVT, selective reperfusion is essential. The OPTIMISTmain (Optimal Post rtPA-IV Monitoring in Ischaemic Stroke Trial—main phase)^112^ showed that low-intensity monitoring after IVT in MIS (NIHSS score <10) was likely noninferior to standard monitoring, supporting less resource-demanding post-IVT care. Locally adapted protocols should weigh symptom severity, comorbidities, and imaging findings to guide management. With uncertain IVT/EVT benefits, DAPT with aspirin–clopidogrel remains the most feasible option. Strengthening health systems through workforce training, efficient resource use, and standardized care is crucial to improve MIS outcomes despite constraints.

## Conclusions

The management of MIS, especially in patients without disabling symptoms or with LVO, remains complex and evolving. The main challenge lies in balancing the generally mild course of MIS with the risk of early deterioration or recurrence. Conventional scales may overlook subtle yet clinically relevant deficits, complicating treatment choices. IVT, EVT, DAPT, and anticoagulation—alone or combined—constitute the current therapeutic spectrum, with indications tailored to each scenario. Strategies vary across regions, and in low-resource settings, prioritization based on severity and cost-effectiveness may be required. Reperfusion decisions should be individualized, considering cause, perfusion deficit, and risk of progression. Trials such as PRISMS,^40^ ARAMIS,^17^ and TEMPO-2^7^ consistently support antiplatelet therapy over IVT in nondisabling MIS, offering clearer guidance for this subgroup. However, uncertainty persists for MIS with LVO, where evidence remains limited. Further well-powered multicenter RCTs are needed to identify predictors of poor outcomes and refine management. Traditional measures like the mRS may miss subtle but meaningful effects. To address this, the COSMOS (Clinical Outcome Score for Minor Stroke)^113^ has been developed—a multidimensional tool assessing motor, cognitive, fatigue, and quality-of-life domains. By capturing these nuanced outcomes, COSMOS may improve clinical decision-making, identify patients for targeted rehabilitation, and enhance evaluation of treatment effects in research.

## ARTICLE INFORMATION

### Author Contributions

Dr Sacco conceived and designed the study and drafted the manuscript. Drs De Santis and Foschi contributed to drafting. All authors revised and approved the final version.

### Sources of Funding

None.

### Disclosures

Dr Coutts reports compensation from Boehringer Ingelheim. Dr Fischer reports support from the Swiss National Science Foundation and Swiss Heart Foundation; research grants from Medtronic, Stryker, Rapid Medical, Penumbra, Phenox, Boehringer Ingelheim; and consultancies and advisory roles with multiple companies. Dr Khatri reports compensation from Translational Sciences for other services; compensation from Silvercreek; and grants from Johnson & Johnson Health Care Systems Inc. Dr Pontes-Neto reports compensation from Bayer for other services and compensation from Boehringer Ingelheim for consultant services. Dr Nguyen reports compensation from Medtronic for consultant services and compensation from Aruna for consultant services. Dr Sandset reports compensation from Bristol-Myers Squibb for other services. Dr Tsivgoulis reports grants from Shire; grants from Genesis Pharma; grants from Allergan; and grants from Amicus Therapeutics Inc. Dr Nasreldein reports advisory fees from Boehringer Ingelheim, Allergan, and Viatris. Dr Sacco reports consultancy or speaker fees from Novartis, Novo Nordisk, Boehringer Ingelheim, Teva, Allergan, Pfizer, Abbott, Lundbeck, AstraZeneca, and Eli Lilly. Dr Turc reports consulting for AI-Stroke, Neurologica, and lectures for Guerbet France.

The other authors report no conflicts.

### Supplemental Material

Tables S1–S3

## Supplementary Material


