# Supplementary material for: Acute Treatment of Disabling and Nondisabling Minor Ischemic Stroke: Expert Guidance for Clinicians
Source: Stroke. 2025 Dec 11;57(2):549–59. doi: 10.1161/STROKEAHA.125.053504 (PMC12829504; doi:10.1161/STROKEAHA.125.053504)
Supplement: Supplementary file 1 [file str-57-549-s001.pdf]

SUPPLEMENTAL MATERIAL

Table S1. Summary of evidence: acute disabling minor ischemic stroke without large vessel occlusion, eligible for intravenous thrombolysis

	Author (Study)	Design	Population	Intervention /Comparator	Outcomes
**Alteplase (TPA)**	Emberson et al., 2014 (45) .	Individual-level meta-analysis including 9 RCTs	n=6756 patients, 666 (10%) with NIHSS 0–4; all trials included only functional relevant deficits, except for one trial allowing also non-disabling symptoms.	TPA vs placebo.	IVT resulted in better functional outcome (mRS 0–1 at 3–6 months) with no significant interaction between baseline NIHSS and treatment effect (p for interaction = 0.06). In NIHSS 0–4 subgroup the OR was 1.48 (95% CI 1.07–2.06),
**Tenecteplase (TNK)**	Alamowitch et al., 2023 (59)	Random-effects meta-analysis of RCTs	n=2197 patients: 1118 TNK vs 1079 TPA.	TNK 0.25 mg/kg vs TPA 0.9mg/kg within 4.5 hours of symptom onset.	TNK demonstrated its non-inferiority to TPA (OR 1.17 [95%CI: 0.98–1.39; $p = 0.08$; $I^2 = 0\%$] in broad stroke population.
	Palaiodimou et al., 2024 (60)	Metanalysis including 11 RCTs	n=3788 TNK vs 3757 TPA.	TNK 0.25 mg/kg vs TPA 0.9mg/kg within hours of symptom onset.	TNK may be superior in achievement of excellent functional outcome (mRS 0–1) ([RR] 1.05, 95% CI 1.01-1.10; $p = 0.012$; $I^2 = 0\%$), reduced disability (≥1-point reduction in mRS scores), with no increase in sICH and similar good functional outcome.
**IVT - extended time window**	Coutts et al., 2024 (7) (TEMPO-2)	RCT	n=886 patients with MIS and an arterial occlusion in the extended window.	TNK treatment compared to non-thrombolytic standard care (within 12 hours of last seen well).	Primary outcome: return to baseline functioning (72% vs 75%, RR 0.96; 95% CI 0.88-1.04, p=0.29). No benefit and potential harm (death in 5% vs 1%, HR 3.8; 95% CI 1.4-10.2, p=0.0085), as evidenced by higher rates of sICH and all-cause mortality.
	Yan et al. (63) (EXPECTS)	RCT	n=117 TPA vs 117 standard care. Posterior circulation stroke without extensive early ischemic changes on CT and no planned thrombectomy.	TPA 0.9mg/kg within 4.5–24 hours of symptom onset vs standard of care.	Higher functional independence (mRS 0-2) at 90 days (9.6% vs. 72.6%; adjusted RR, 1.16; 95% CI, 1.03 to 1.30; p = 0.01),<br>The benefit was observed even in patients with mild clinical deficits: low NIHSS (median 3 [IQR 2-6]) IVT group and 3 [IQR 1–6] in the standard care group.
	Ma et al., 2019 (62) (EXTEND)	RCT	n=225 patients (113 TPA vs 112 placebo) guided by perfusion imaging.	TPA 0.9 mg/kg up to 9 hours of symptom onset vs standard of care.	A comparable benefit over placebo in achieving functional independence (mRS 0–1 at 90 days) irrespective of baseline stroke severity below or above NIHSS 10 (adjusted RR 1.47; 95% CI, 0.98–2.20; interaction p=0.530).

Abbreviations. MIS Minor ischemic stroke, DAPT Dual antiplatelet therapy, SAPT Single antiplatelet therapy; TPA-tissue plasminogen activator-alteplase, TNK Tenecteplase, ASA aspirin , IVT intravenous thrombolysis, LD loading dose, NIHSS National Institutes of Health Stroke Scale; mRS modified Rankin scale, RCT Randomized controlled trial, IQR interquartile range, OR odds ratio, CI confidence interval.

Table S2. Summary of evidence: acute disabling or non-disabling minor ischemic stroke, not eligible for acute reperfusion therapies

Type of evidence	Author (Study)	Design	Population	Intervention /Comparator	Outcomes
**Non-cardioembolic MIS**	Kennedy et al., 2007 (FASTER) (64)	RCT	n=396 patients with MIS: NIHSS ≤ 3* ABCD2≥4, within 24 hours after the onset.	ASA: LD 162 mg then 81 mg/d, Clopidogrel: LD 300 mg then 75 mg/d for 90 days.<br><br>Comparator: ASA LD 162mg then 81 mg/d and placebo.	Primary: 90-day any recurrent stroke (ischemic or hemorrhagic) 7.1% in DAPT vs 10.8% in SAPT.
	Wang et al., 2013 (CHANCE) (11)	RCT	n=5170 patients with MIS: NIHSS ≤ 3* ABCD2≥4, within 24 hours of symptoms onset.	ASA: 75-300 mg LD followed by 75 mg daily Clopidogrel: 300 mg LD followed by 75 mg daily for 21 days then ASA on days 22 through 90.<br><br>Comparator: ASA LD 75-300mg then 75mg/d and placebo.	Primary: 90-day new stroke event (ischemic or hemorrhagic): 8.2% in DAPT vs 11.7% in SAPT. Moderate-to-severe bleeding events***: 0.3% DAPT vs. 0.3% in SAPT.
	Johnston et al., 2018 (POINT) (12)	RCT	n=4881 patients with MIS: NIHSS ≤ 3* ABCD2 ≥4, within 12 hours of symptoms onset.	ASA: 50- 325 mg daily. Clopidogrel: 600-mg LD followed by 75 mg daily for 90 days.<br><br>Comparator: ASA 50-325mg/d and placebo.	Primary : composite of ischemic stroke, myocardial infarction, or death from ischemic vascular causes (5.0% in DAPT vs. 6.5% in SAPT group). Major hemorrhage: 0.9% in DAPT vs. 0.4% in SAPT group.
	Johnston et al., 2020 (THALES) (6)	RCT	n=11 016 patients with MIS: NIHSS ≤5** ABCD2 ≥6 or symptomatic intra/extracranial arterial stenosis*, within 24 hours of symptom onset.	ASA: 300- 325 mg LD followed by 75-100 mg daily Ticagrelor: 180 mg LD followed by 90 mg twice a day for 30 days.<br><br>Comparator: ASA 300- 325 mg LD followed by 75-100 mg daily and placebo.	Primary: composite of stroke (ischemic stroke, hemorrhagic stroke) or death (5.5% in DAPT vs 6.6% in SAPT). Severe bleeding: 0.5% in DAPT vs. 0.1% in SAPT.
	Gao et al., 2023 (INSPIRES) (66)	RCT	n=6 100 patients with MIS: NIHSS ≤5 ABCD2≥4. Presumed Large artery atherosclerosis origin, within 72-hours of symptom onset.	ASA: LD 100 to 300 mg followed by 100 mg daily Clopidogrel: LD 300 mg followed by 75 mg/d.<br><br>Comparator: ASA: LD 100 to 300 mg followed by 100 mg daily and placebo.	Primary: any new stroke (ischemic or hemorrhagic) within 90 days. 7.3% in DAPT vs. 9.2% in SAPT group. (HR 0.79; 95% CI 0.66 to 0.94; p=0.008). Bleeding: 0.9% in DAPT vs 0.4% in SAPT
	Wang et al., 2021 (CHANCE-2) (69)	RCT	n=6412 patients with MIS: NIHSS ≤ 3* ABCD2≥4, within 24 hours of symptom onset, in CYP2C19 loss-of-function carriers.	Ticagrelor (180 mg LD followed by 90mg twice daily), ASA LD 75-300mg followed by 75mg daily for 90 days.	Primary outcome: a greater reduction in 90-day stroke risk in ticagrelor (6.0%) compared to clopidogrel and ASA (7.6%) (HR, 0.77; 95% CI, 0.64–0.94; p=0.008), with no significant difference in major bleeding

				Comparator: Clopidogrel 300mg LD, followed by 75 mg, ASA LD 75-300mg followed by 75mg daily.	events. Both study arms were associated with the same 0.3% rates of moderate-to-severe bleeding,
	Bhatia et al., 2021 (65)	Meta-analysis	n=21 459 patients with MIS.	DAPT vs SAPT	DAPT significantly reduced: recurrent stroke (relative risk [RR], 0.76 [95% CI, 0.68–0.83]; p<0.001), MACE (RR, 0.76 [95% CI, 0.69–0.84]; p<0.001) and recurrent ischemic events (RR, 0.74 [95% CI, 0.67–0.82]; p<0.001), but increased the major bleeding risk (RR, 2.22 [95% CI, 1.14–4.34]; p=0.02).
	De Matteis et al., 2023 (READAPT) **(**67)	Real-world study	n=1920 patients with MIS.	DAPT	Despite broader application than RCTs, similar effectiveness/safety (with primary effectiveness occurring in 3.9% and primary safety outcomes in 0.6%). Cerebrovascular ischemic recurrences occurred in 3.3%, intracranial hemorrhage in 0.2%, and bleeding events in 2.7%; mortality due to vascular causes was 0.2%.
	Lim et al., 2024 (72)	Bayesian meta-analysis	n=22203 patients with MIS: 13995 (63.0%) were in DAPT and 8208 (37.0%) in aspirin.	DAPT vs SAPT (clopidogrel or ticagrelor + ASA)	The combination with ticagrelor ranked best for benefit and the overall positive results lost significance in sensitivity analyses, so results need cautious interpretation.
**Cardioembolic MIS**	Goeldlin et al., 2024 (ELAN) (79)	RCT (post-hoc analysis)	n=1962 MIS: defined by an infarct size <1.5 cm in the anterior or posterior circulation territory.	Early DOAC (≤48 hours in MIS) vs late DOAC (>3-4 days)	Early anticoagulation (<48h from symptoms onset) resulted in lower incidence of the primary outcomes: 2.7% in early vs 3.0% in late (OR 0.89; 95% CI, 0.38-2.10).
	Dehbi et al., 2025 (CATALYST) (80)	Individual level meta-analysis of RCTs	n=5441 patients with cardioembolic stroke (median NIHSS = 5).<br><br>Data from the RCTs TIMING(71), ELAN(72) , OPTIMAS(73) and START(74): 4075 (75%) patients had mild-to-moderate stroke (NIHSS 0–10).	Early DOAC (≤4 days) vs late DOAC (>4 days)	Primary composite outcome (recurrent ischemic stroke, sICH, or unclassified stroke) within 30 days: 2.1% in DOAC early vs 3.0% (OR 0·70, 95% CI 0·50–0·98, p=0·039). Consistent benefit across key prespecified clinical subgroups including MIS defined with baseline NIHSS-score of 0-4.

Abbreviations. MIS Minor ischemic stroke, DAPT Dual antiplatelet therapy, SAPT Single antiplatelet therapy, ASA aspirin, LD loading dose, NIHSS National Institutes of Health Stroke Scale, RCT Randomized controlled trial, DOAC direct oral anticoagulant, IQR interquartile range, OR odds ratio, RR risk ratio, CI confidence interval. *included also high-risk TIA with ABCD2≥4, **included also high-risk TIA with ABCD2 ≥6 or symptomatic intra/extracranial arterial stenosis *** according to the Global Utilization of Streptokinase and Tissue Plasminogen Activator for Occluded Coronary Arteries (GUSTO) classification.

Table S3. Summary of evidence: acute non-disabling minor ischemic stroke without large vessel occlusion eligible for intravenous thrombolysis

Type of evidence	Author (study)	Design	Population	Intervention/Comparator	Outcome
**IVT vs antiplatelet therapy**	Khatri et al., 2018 (40) (PRISMS)	RCT	n=313; acute mis NIHSS 0–5 and non-disabling judged deficits (with no impact on performing basic activities of daily living or work).	TPA (0.9 mg/kg, within 3h) + oral placebo withing 3 hours from onset vs ASA 325 mg + IV placebo.	No significant difference in 90-day favorable outcome (mRS 0-1): 78.2% (TPA) vs 81.5% (ASA); sICH in 5 (3.1%) treated with TPA vs 0 with ASA ; No benefit; Early terminated.<br><br>Bayesian post hoc analysis estimated a posterior probability of 1.9% that alteplase would achieve an absolute treatment benefit exceeding 6% (unadjusted risk difference, 95% credible interval – 12.2% to 5.5%).
	Chen et al., 2023 (17) (ARAMIS)	RCT	n=760; acute mild non disabling stroke (NIHSS ≤5, with 0–1 on key items: vision, language, neglect, single limb weakness).	DAPT: clopidogrel 300 mg LD and ASA 100mg the first day, followed by clopidogrel 75mg and ASA 100 mg for 12 ± 2 days), followed by SAPT vs TPA administered within 4.5 hours from symptom onset followed by SAPT.	DAPT was noninferior to IVT. Excellent functional outcome (mRS 0-1): 93.8% DAPT vs 91.4% TPA; sICH 0.3% vs 0.9%.
	Xiong et al., 2024 (9) (PUMICE)	RCT	n=236 patients with non-disabling acute MIS (NIHSS ≤5).	IV prourokinase 35 mg bolus, ≤4.5h from onset + antiplatelet vs standard of care antiplatelet alone (single aspirin or clopidogrel or dual).	Prourokinase was not superior to standard care. No difference in excellent functional outcome (mRS 0–1) at 90d: 73.5% vs 81.2%; (risk difference −7.6%, 95% CI −18.3 to 3.0; p=0.16), 2 cases of sICH (1.7%) in prourokinase vs 0 (0%) in standard of care; stopped early for futility.
	Coutts et al., 2024 (7) (TEMPO-2)	RCT	n=300 patients with acute MIS (NIHSS ≤ 5) associated with an intracranial occlusion or focal perfusion abnormality suggesting an occlusion, and for whom IVT was not warranted based on clinical and patient judgement (without further specifications).	TNK 0.25 mg/kg ≤12h from onset vs non-thrombolytic standard of care.	No evidence of benefits from IVT. Primary outcome (return to baseline functioning mRS): no benefit from IVT (72% TNK vs 75% control group, n.s.); Higher mortality (5% vs 1%); Higher sICH (2% vs <1%); stopped early for futility.
	Lun et al., 2024 (84)	Bayesian network meta-analysis	n=5897 patients from 4 RCTs and 2 observational studies.	TPA (alteplase) vs DAPT (clopidogrel + aspirin).	DAPT higher odds of excellent functional outcome (mRS 0–1) compared to TPA (OR 1.52, 95% CI

				1.09–2.35). Lower odds of symptomatic intracranial hemorrhage than TPA (OR = 0.14, 95% CrI, 0.03–0.91).
Doheim et al., 2025 (85)	Meta-analysis	n=3364 patients from 4 RCTs with acute MIS.	IVT vs nonthrombolytic standard of care.	IVT offered no functional benefit; potentially higher sICH and mortality.

Abbreviations. MIS Minor stroke, DAPT Dual antiplatelet therapy, SAPT Single antiplatelet therapy, ASA aspirin, TPA alteplase, TNK Tenecteplase, LD loading dose, NIHSS National Institutes of Health Stroke Scale, mRS modified Rankin scale, sICH symptomatic intracranial hemorrhage, LVO large vessel occlusion, RCT Randomized controlled trial, OR odds ratio, RR risk ratio.
